# Rapid identification of pneumococci, enterococci, beta-haemolytic streptococci and *S. aureus* from positive blood cultures enabling early reports

**DOI:** 10.1186/1471-2334-14-146

**Published:** 2014-03-19

**Authors:** Marie C Larsson, Ewa Karlsson, Hanna Woksepp, Kerstin Frölander, Agneta Mårtensson, Foad Rashed, Wistedt Annika, Thomas Schön, Lena Serrander

**Affiliations:** 1 Division of Clinical Microbiology, Department of Clinical and Experimental Medicine, Linköping University, Linköping, SE 581 85, Sweden; 2 Division of Microbiology, Kalmar County Hospital, Kalmar, SE 391 26, Sweden; 3 Division of Infectious Diseases, Kalmar County Hospital, Kalmar, SE 391 26, Sweden; 4 Division of Infectious Diseases, Department of Clinical and Experimental Medicine, Linköping University, Linköping, SE 581 85, Sweden

**Keywords:** Sepsis, Microbiology, Rapid diagnostics, Bacteraemia, Agglutinations, Gram-positive

## Abstract

**Background:**

The aim of this study was to evaluate diagnostic tests in order to introduce a diagnostic strategy to identify the most common gram-positive bacteria (pneumococci, enterococci, β-haemolytic streptococci and *S. aureus*) found in blood cultures within 6 hours after signalling growth.

**Methods:**

The tube coagulase test was optimized and several latex agglutination tests were compared and evaluated before a validation period of 11 months was performed on consecutive positive blood culture patient samples from Kalmar County Hospital, Sweden.

**Results:**

During the validation period 150 (91%) of a total of 166 gram-positive cocci (119 in clusters, 45 in chains or pairs and 2 undefined morphology) were correctly identified as *S. aureus*, CoNS, Pneumococci, Enterococci or group A streptococci (GAS), group B streptococci (GBS), group G streptococci (GGS) within 6 hours with a minimal increase in work-load and costs. The remaining samples (9%) were correctly identified during the next day. No samples were incorrectly grouped with this diagnostic strategy and no patient came to risk by early reporting.

**Conclusion:**

A simple strategy gives reliable and cost-effective reporting of >90% of the most common gram-positive cocci within 6 hours after a blood cultures become positive. The high specificity of the tests used makes preliminary reports reliable. The reports can be used to indicate the focus of infection and not the least, support faster administration of proper antimicrobial treatment for patients with serious bacterial infections.

## Background

Sepsis is a challenge in medicine worldwide with high mortality if not treated rapidly and adequately [1-3]. Faster preliminary reports from the microbiology laboratories could not only help when empirical treatment fails, but also serve to narrow treatment when the preliminary report supports the clinical picture. Molecular methods, mass spectrometry and fluorescence in situ hybridisation such as real-time PCR, MALDI-TOF and PNA FISH for rapid identification of positive blood cultures are attractive improvements [4-6]. However they can be complemented with inexpensive rapid tests, as has been pointed out earlier [7]. Moreover, some of the species identified here e.g. pneumococci, viridans streptococci and staphylococci has proven to be identified with less accuracy when diagnosed with e.g. MALDI-TOF [8-10] and the preparation time from blood cultures is longer than for some of the tests used in this study. A recent study shows that the identification rate for streptococci for MALDI-TOF from blood cultures can be as low as 22%, but increased in combination with Gram stain [11]. Gram-positive cocci (GPC) in clusters have also been reported to be more difficult to identify with MALDI-TOF [12].

The purpose of this study was to evaluate and set up a diagnostic approach of positive blood cultures to enable a faster preliminary reporting of a group of the most common GPC, which would be followed by confirming and complementary reports later that same day. The goal was mainly to discriminate among β-haemolytic, non-β-haemolytic streptococci, enterococci, *S. aureus* and coagulase-negative staphylococci (CoNS). This strategy does not aim to identify all species of gram-positive bacteria, rather a complement than a substitution to other laboratory routines such as MALDI-TOF or other identification methods. Evaluation was performed with two different blood culture systems; BACTEC 9240 (Becton Dickinson, Sparks, MD, USA) and BacT/Alert (bioMérieux, Marcy l’Etoile, France) are used. BACTEC is used in Linköping and BacT/Alert in Kalmar.

We have also retrospectively investigated if the time from incubation start until signalled growth of blood cultures could be of value in the discrimination process. The work was carried out in two phases. The first evaluation phase involved comparing latex agglutination tests directly on material from positive blood culture bottles, previously shown to be reliable when performed on grown colonies [13,14]. Also the tube coagulase test (TCT) was evaluated in regard to blood volume and plasma species used, which showed to have importance. A second validation phase, based on findings of the first phase, was then performed where a diagnostic strategy was applied on consecutive patient samples.

## Methods

### Samples for method validation

Clinical strains, primarily analyzed according to conventional identification strategies such as culture on selective agar (esculin, DNAse), biochemical testing (optochin, PYR, bacitracin, CAMP) including analytical profile index (API) and complementary agglutination tests were used. For evaluation experiments, these strains were inoculated at the approximate amount of 100 CFU in former culture-negative blood culture bottles. The bottles used were aerobic Plus/F for the BACTEC system and BacT/Alert aerobic bottle (FA) for the BacT/Alert system.

Material from inoculated, previously negative blood cultures bottles and henceforth called blood culture material.

### Setting and patient samples

The major part of the rapid method testing was performed, at the Division for Clinical Microbiology, Linköping University Hospital, on material from inoculated negative BACTEC blood culture bottles and agar plates. When stated that BacT/Alert was used, experiments were performed at the division for Clinical Microbiology in Kalmar.

The consecutive samples evaluated in this study were 166 patient blood cultures containing GPC from Kalmar County Hospital (catchment population 233 000) where 4 300 pairs of blood culture bottles are collected per year, of which 18% are positive. GPC are found in roughly half of the positive blood cultures. The species distribution was for the year of 2009: *S. aureus* 30%, CoNS 20%, Pneumococci 14%, Enterococci, 14%, group A streptococci 4%, group B streptococci 2%, other streptococci 8%, other GPC 8% (CoNS singlets excluded as probable contaminations).

Samples were taken as part of standard patient care. Ethical approval was obtained from the Regional Ethical Review Board in Linköping, number M17-09.

### Tube coagulase test (TCT)

TCT was performed with 25 μL, 75 μL, 200 μL and 300 μL blood culture material and 0.5 mL of plasma; from rabbit (BBL Coagulase plasma with EDTA, Becton Dickinson, USA), horse (Swedish National Veterinary Institute, Uppsala, Sweden) and human (Division of Transfusion Medicine, Linköping University Hospital, Linköping, Sweden) to find the optimal blood volume and plasma type. The samples were incubated at 36°C and examined for coagulation every 15 minutes during 4 hours and finally at 24 h. *Staphylococcus aureus* ATCC 25923 and *Staphylococcus saprophyticus* CCUG 3706 isolates were used as positive and negative controls.

### Staphylococcal agglutination

Three Staphylococcal agglutination kits; Prolex™ Staph latex kit (Pro-lab Diagnostics, Richmond Hill, ON, Canada), Slidex Staph kit (bioMérieux, Marcy l’Etoile, France), Prolex™ Staph Xtra Latex kit (Pro-lab Diagnostics, Richmond Hill, ON, Canada) were evaluated at the microbiology laboratory of Linköping University Hospital. The tests were performed on material from blood culture bottles inoculated with *S. aureus* (2 drops were used, which corresponds to the volume used for colony testing according to the manufacturers recommendation) and colonies grown 4–8 hours on blood agar plates from the same blood cultures according to the manufacturer’s instructions.

### Pneumococcal agglutination

The Oxoid Dryspot Pneumo Test (Oxoid, Hampshire, UK) was performed with inoculated samples incubated in the BACTEC system the microbiology laboratory of Linköping University Hospital both on blood culture material and colonies grown 4–8 hours on blood agar plates according to manufacturer’s instructions except that the centrifugation step was left out.

### Streptococcal agglutination

Prolex Streptococcal grouping test (Pro-lab Diagnostics, Richmond Hill, ON, Canada) was evaluated both at the microbiology laboratory of Linköping University Hospital and Kalmar County Hospital and compared to the streptococcal grouping test used in the routine at each laboratory, Phadebact Streptococcus test (Bactus AB, Huddinge, Sweden) was evaluated in Linköping and Streptex Test kit (Remel Inc. Lenexa, KN, USA) in Kalmar. The kits were tested on bacterial colonies according to manufacturers’ instructions and on blood culture material by using two drops of blood material to one drop of test reagent without prior pre-treatment. Two drops were chosen because it corresponds to the volume used to mix colonies in when tested as described in the manufacturer’s instruction.

The agglutinations from colonies were complemented with PYR-test (L-pyrrolidonyl-ß-naphthylamide, O.B.I.S.-PYR, Oxoid LTD, Basingstoke, England) for discriminating GAS and enterococci from other streptococci [15].

### Time until signalled growth

Time to signalled growth (the time evolved from the start of the incubation until the blood culture system signalled growth), was analysed retrospectively for 360 patient samples positive for GPC incubated in BACTEC, in the Linköping laboratory. Analyses involved retrieving the sensitivity, specificity and positive and negative predictive values of the cut-off time points suggested by Martínez et al. [16].

### Consecutive samples

**Figure 1:**
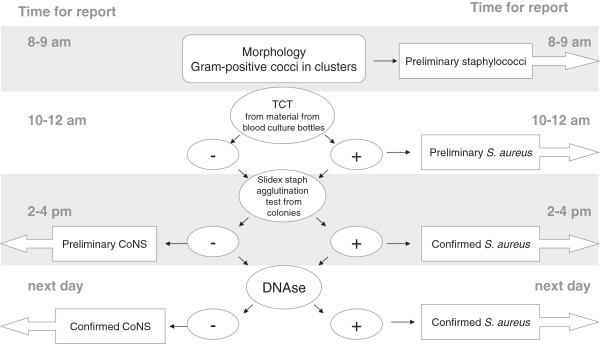
**Flowchart for identifying gram-positive cocci in clusters.** After the Gram staining and subculture on blood and chocolate agar, tube coagulase tests (TCT) were performed by adding 75 μL material from positive blood culture bottles to 0.5 mL horse plasma and analysed for coagulation every 15 min until 4 hours and again after 24 h. When colonies were visible (after 4–6 hours), agglutination tests were performed. The following day complementary tests were analysed. The consecutive patient samples were analysed and reported according to this chart. Abbreviations in figure: TCT: tube coagulase test, CoNS: Coagulase-Negative Staphylococci.

Our strategy for GPC in clusters was to immediately culture the blood bottle material from positive blood culture bottles onto solid blood agar plates and to initiate a TCT. During the time it takes for *S. aureus* to produce a coagulate in the TCT, colonies will shortly appear on the solid agar, which can be used for a latex agglutination test. The process is illustrated in Figure 1.

**Figure 2:**
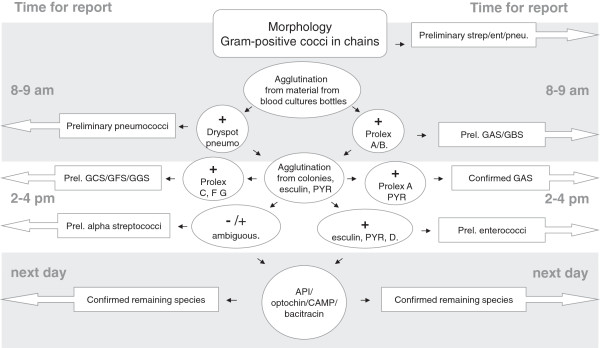
**Flowchart for identifying cocci in chains.** After morphology was determined by Gram staining, subcultures were performed on blood, chocolate and esculin agar plates. Samples from positive blood culture bottles were then tested first with Oxoid Dryspot Pneumo Test and then Prolex Lancefield agglutination tests. When colonies were visible, agglutinations were performed again and esculin positivity and well as PYR tests (positive for GAS and enterococci) were included in the results. The following day complementary tests were read, e.g. API for ambiguous gram-positive cocci, optochin /bacitracin susceptibility for pneumococci and group A streptococci respectively and CAMP for Group B streptococci, which is positive by detecting haemolysis by an indicator strain of *S. aureus*, which takes place in the presence of a factor produced by GBS. Abbreviations in the figure: Prel.: preliminary, strep: streptococci, ent: enterococci, pneu.: pneumococci, GAS/GBS/GCS/GFS/GGS: group A/B/C/F/G streptococci, PYR: L-pyrrolidonyl-ß-naphthylamide-test, CAMP: a test for GBS identification, API: analytical profile index, a commercial phenotypic test for species determination.

For streptococci the strategy was to identify blood cultures containing beta-haemolytic streptococci, enterococci and pneumococci by using agglutination kits directly on material from positive blood culture bottles; with repeated testing on colonies after 6 hour incubation on solid blood agar. Prolex Streptococcal grouping kit, Oxoid Dryspot Pneumo Test and subcultures on blood, chocolate and esculin agar was performed on all GPC in pairs or chains. The process is illustrated in Figure 2.

This strategy was applied at the microbiology laboratory of Kalmar County Hospital, Sweden during March 1st 2009 to January 31st 2010. Blood cultures, positive weekdays before 8 am, showing GPC, were included. Blood cultures from 691 patients were positive for bacterial growth during this time period. Out of these, 164 contained GPC and were subjected to the processes and reported as shown in Figures 1 and 2 in addition to the conventional procedure used in the laboratory. Two independent species-specific tests were always used for species determination.

### Statistics

Sensitivity and specificity are presented as percentage of results obtained by golden standard methods. Chi-square tests were carried out for non-metrical results. Significance levels are shown as stars (p < 0.001 = ***, p < 0.01 = **, p < 0.05 = *, n.s. = non-significant).

## Results

### Testing of purified clinical isolates

#### Evaluation of the tube coagulation test (TCT)

**Figure 3:**
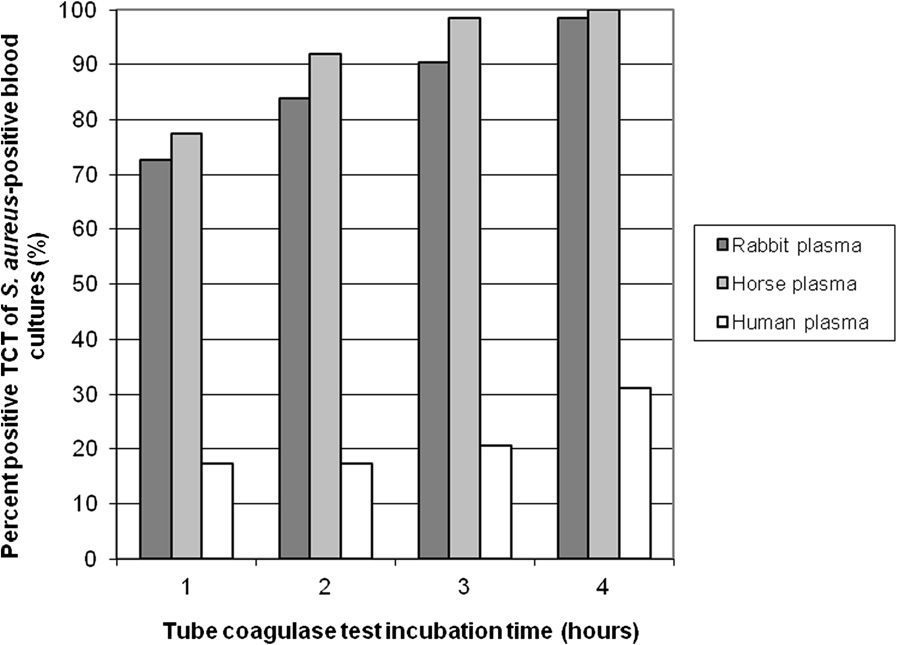
**The sensitivity of the tube coagulase tests (TCT) for *S. aureus *varies with plasma species used in the test.** Twelve *S. aureus* strains were inoculated in previously negative blood culture bottles, incubated and when signalling positive subjected to tube coagulase test (TCT) by addition of 75 μL material from inoculated blood cultures bottles positive for *S. aureus* to 0.5 mL plasma of three species; rabbit (n = 62), horse (n = 62) and human (n = 29). The tubes were incubated at 36°C and observed for coagulation every 15 minutes during the first four hours and then after 24 hours of incubation. The figure shows the amount of positive tests within the different time intervals in percent of total bottles incubated with *S. aureus.*

**Figure 4:**
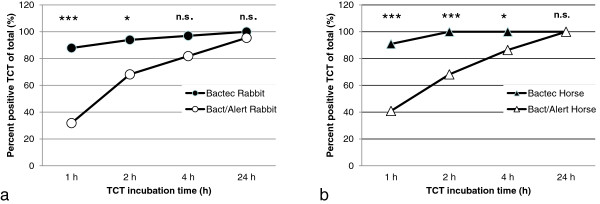
**The rapidity of positive tube coagulase test (TCT) differs between blood culture systems.***S. aureus*-inoculated bottles were incubated in BACTEC (filled symbols) and BacT/Alert (empty symbols) respectively and when signalling positive were analysed for time to positivity in the TCT. The results using rabbit plasma is shown in Figure 4**a** and horse plasma in Figure 4**b**. Statistics were performed using Chi square tests comparing Bactec and BacT/Alert blood culturing systems. Significance levels are shown as stars (p < 0.001 = ***, p < 0.01 = **, p < 0.05 = *, n.s. = non-significant).

Rabbit and horse plasma showed positive results within 4 hours for 61/62 and 62/62 of the *S. aureus* inoculated samples respectively with the blood volume of 75 μL (Figure 3). All samples with *S. aureus* were positive after 24 hours when using horse or rabbit plasma. Human plasma was shown to be ineffective in the TCT, only 31% of samples with *S. aureus* showed a positive result after 4 h incubation. Volumes of material from positive blood culture bottles ranging from 25 to 300 μL were added to 0.5 mL plasma from horse, rabbit or human origin. The highest sensitivity, without affecting specificity was obtained by adding 75 to 200 μL of blood culture material positive for *S. aureus.* The mean time to coagulation was 95 min for rabbit plasma and 73 min for horse plasma*.* Lower volume e.g. 25 μL (approximately 1 drop of blood) showed inferior sensitivity. There was a significant difference in time to a positive TCT between BACTEC and BacT/Alert, to the advantage of BACTEC (Figure 4). Inoculated and fresh patient blood culture samples with CoNS were tested as negative controls with horse and rabbit plasma, all being negative throughout the testing period, giving a specificity of 100% for all plasma types. Additionally, *Staphylococcus saprophyticus* CCUG 3706 and *Staphylococcus aureus* ATCC 25923 were negative and positive controls respectively at each testing occasion.

### Evaluation of agglutination for *Staphylococcus spp.*

Out of the inoculated samples of *S. aureus* (n = 20) tested directly from positive blood culture material, none (0/20) of the samples agglutinated with any of the three staphylococci latex-kits. Visible colonies were observed in all cases of *S. aureus* following 4–6 h incubation on blood agar plates. All *S. aureus* samples from colonies on agar plates were agglutination-positive for all three latex-kits evaluated. The CoNS samples tested showed negative test results both when tested directly on blood material and on colonies after incubation on agar plates (n = 20).

### Evaluation of agglutination of β-haemolytic streptococci and enterococci

When used directly on material from positive blood cultures, the sensitivity for the Oxoid Dryspot Pneumo Test was 77% (17/22). The remaining five samples with pneumococcal morphology were positive when retested with cultured colonies the same day. Non-pneumococcal inoculated samples (n = 33) incubated in BACTEC system consisting of *E. faecalis* (n = 9), *E. faecium* (n = 5), *Streptococcus pyogenes* (n = 2), *Streptococcus agalactiae* (n = 2), *S. oralis* (n = 4), *S. sanguis* (n = 4), *S. intermedius* (n = 1) and *S. mitis* (n = 4), *S. constellatus* (n = 1), *S. dysgalactiae* (n = 1), were tested with negative results from both material from positive blood culture bottles and outgrown colonies.

### Agglutination of β-haemolytic streptococci and enterococci

Inoculated samples in the two blood culturing systems were tested with two kits each. Prolex Streptococcal grouping latex kit and Phadebact Streptococcus Test were performed on samples incubated in BACTEC with the following isolates: group A streptococci (*S. pyogenes*, n = 11), group B streptococci (*S. agalactiae*, n = 12), group C streptococci (*S. intermedius* (n = 1), *S. dysgalactiae* ssp. *dysgalactiae* (n = 1), *S. dysgalactiae* ssp*. equisimilis* (n = 1)), group D streptococci (*E. faecalis* (n = 7), *E. faecium* (n = 9)), group F streptococci (*S. anginosus* (n = 2), *S. intermedius* (n = 2), *S. mitis* (n = 1), *S. constellatus* (n = 1)), group G streptococci (*S. dysgalactiae* ssp*. dysgalactiae* (n = 4), *S. dysgalactiae* sp. (n = 1), *S. dysgalactiae* ssp*. equisimilis* (n = 6), *S. constellatus* (n = 1), *S. canis* (n = 1)) and as negative controls *S. pneumoniae* (n = 5), CoNS (n = 2) and *S. aureus* (n = 3). Prolex Streptococcal grouping latex kit and Streptex Kit were tested on the following samples incubated in BacT/Alert: group A streptococci (*S. pyogenes,* n = 3), group B streptococci (*S. agalactiae,* n = 4), group C streptococci (n = 4), *E. faecium* (n = 4), *E. faecalis* (n = 4), group G streptococci (n = 3) and as negative controls *S. milleri* (n = 1), *S. bovis* (n = 1), *S. aureus* (n = 1) and *E. coli* (n = 1). The sensitivity and specificity of the different streptococcal grouping kits used on blood culture bottles are shown in Table 1.

**Table 1:** Agglutination results for beta-haemolytic streptococci and enterococci in two different blood culturing systems

	**Prolex streptococcal latex kit**		**Phadebact streptococcus test**		**Streptex kit**	
	**Sensitivity**	**Specificity**	**Sensitivity**	**Specificity**	**Sensitivity**	**Specificity**
BACTEC	93%	99%	98%	9%	Not determined	
BacT/Alert	95%	100%	Not determined		86%	100%

False negative results were shown with Prolex Streptococcal grouping latex kit for three *E. faecium* and one strain of Group F streptococci incubated in BACTEC and one strain of Group C streptococci incubated in BacT/Alert. Four *S. pneumoniae* incubated in BACTEC, showed positive results with the group C reagent, similar to what has been shown previously [17]. All four of these were positive in the Oxoid Dryspot Pneumo Test agglutination and found to be true pneumococci the next day.

### Time to positive signal

Table 2 shows the sensitivity, specificity and predictive values for the retrospective blood cultures samples which we evaluated to according to the cut-off values for discrimination between *S. aureus* and CoNS and for separation of β-haemolytic streptococci, pneumococci and enterococci from other viridians streptococci [16]. Although Martinez’s et al. chose to define enterococci as belonging to the slow growing group of GPC in chains, they were here grouped together with β-haemolytic streptococci and pneumococci, since enterococci in our study had significantly shorter time to positive signal.

**Table 2:** Time to positive signal from retrospectively analysed blood cultures containing gram-positive cocci

	**Cut-off**	**Sensitivity (%)**	**Specificity (%)**	**Positive predictive value (%)**	**Negative predictive value (%)**
*S. aureus* vs. CoNS, cut off time points	≤7 h	9	100	100	63
	≤23 h	86	59	58	87
β-haemolytic, pneumococci and enterococci vs. viridians group	≤6 h	9	100	100	14
	≤21 h	94	50	94	50

Consecutive patient blood cultures (n = 360) from a 3 month period were analysed according to time to positive signal (incubation time until positive growth signal), using cut-off time points suggested by Martínez et al. [16] with the modification that enterococci were included in the fast-growing group of streptococci. This analysis was performed in order to evaluate the utility of time to positive signal in the identification process.

### Prospective evaluate of the diagnostic strategy of directly testing consecutive blood cultures growing gram positive cocci

**Figure 5:**
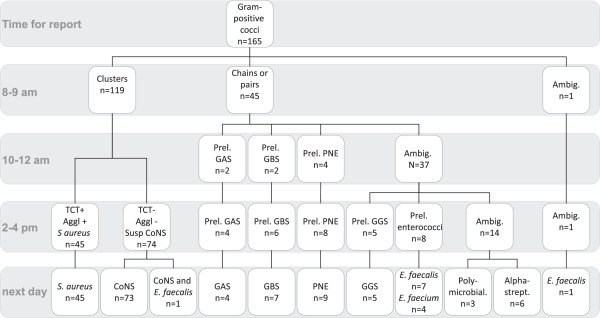
**Species identification and reports given based on rapid testing on consecutive patient samples.** The flowchart approach in Figures 1 and 2 was used to analyse gram-positive samples from patients in Kalmar from March 1st 2009 to January 31st 2010. The chart shows the information given to clinicians at different times. (Prel.: Preliminary, TCT: tube coagulase test, aggl: agglutination, PNE:pneumococci, GAS/GBS/GCS/GFS/GGS: group A/B/C/F/G streptococci, ENT: enterococci, CoNS: coagulase-negative staphylococci, ambig: ambiguous).

Out of the blood cultures showing GPC in clusters (n = 119), 45 of the cultures could be discriminated as *S. aureus* within 6 hours of positive signalling (Figure 5). All of these were confirmed as *S. aureus* the next day, by positive agglutination and positive TCT. The 74 remaining samples were identified as CoNS (n = 73) with agglutination and TCT and the remaining sample was shown to be polymicrobial, containing CoNS and *E. faecalis*.

Of the samples containing GPC in chains or pairs (n = 45) according to direct microscopy, 36 were found to be species which the studied diagnostic approach aims to identify. Three samples were shown to be polymicrobial (*E. faecalis* and *S. aureus*, *E. faecalis* and *Enterobacter cloacae, S. pyogenes* and CoNS) and six samples contained alpha haemolytic streptococci. Preliminary reports were acquired for 22% (8/36) during the morning and for 86% (31/36) during the afternoon. Detailed information on what was reported at different time points is shown in Figure 5.

## Discussion

In this study we show that a rapid diagnostic approach can generate a preliminary species report within 1–6 hours for the majority of the consecutive samples in the prospective validation. These reports were in the majority of cases later confirmed with additional tests including antibiotic susceptibility testing to achieve definite results in final reports. There was very little increase in work-load, mainly shifting the analyses to the day before, since agglutination conventionally often is performed the day after blood cultures become positive.

Before the prospective evaluation we systematically evaluated TCT using plasma from three different species as well as various agglutination tests for the identification of GPC. The fact that TCT is a sensitive, cost-effective and rapid method to differentiate staphylococci has been confirmed in earlier studies [18,19], but here we show that the sensitivity can be further increased by optimization of the volume taken from blood-culture bottles and in the choice of plasma. Horse plasma seemed to give more rapid positivity than rabbit plasma, which is the gold standard method. Human plasma was in our hands neither rapid, nor sensitive enough to be used at all.

Our results suggest that the BACTEC blood culture bottles are more suitable for rapid testing than those from BacT/Alert. Differences between hospitals are less likely since most of the tests were carried out by the same persons at both sites. This may be due to the coal-particles in the BacT/Alert bottles. Since our results were obtained, a new type of BacT/Alert bottles without coal-particle has been developed and they may perform better.

The specificity of the Oxoid Dryspot Pneumo Test and TCT was high when testing directly from blood, whereas the Lancefield agglutination tests should be complemented with further tests or testing on colonies to be reliable, in particular for streptococci other than group A or B. Here the PYR-test was valuable in particular to distinguish enterococci from α-streptococci, which is of high clinical value since the mortality from enterococcal septicaemia is high due to delayed effective antibiotic treatment [20].

Thus, the results from agglutination of GPC in chains directly from blood culture bottles suggest a high positive predictive value, but that the negative results should be re-evaluated from 4–6 hour grown colonies.

Using the time to positive signal of a sample with known gram staining morphology has some supportive value, as previously reported [16].

The species determination is important, particularly for gram-positive bacteria, but the drug susceptibility pattern may also be important although for empirical treatment in the Nordic setting with low vancomycin-resistant enterococci (VRE) and methicillin-resistant *S. aureus* (MRSA) prevalence, the correct identification of enterococci and *S. aureus* is usually enough for selecting the most appropriate initial antibiotic regimen.

In the future we hope that there could be standardized break-points earlier after plating so that disc-diffusion tests could be made directly from positive blood cultures. An alternative strategy may be to use the more expensive E-test [21], which are less sensitive to the size of the inoculum for a few key antibiotics.

It is important to take advantage of all information obtainable for each blood culture as fast as possible and weigh it together to reach reliable species identifications [7]. Earlier reporting can often help to direct attention to the infectious focus if that is not previously known [22].

For some species (e.g. pneumococci, group A streptococci), sufficient identification criteria can be acquired within 2 hours to give physicians preliminary species identifications with more than 90% accuracy, and in some cases more time is required until the identification is more reliable (e.g. alpha streptococci).

Similar strategies have already been published and implemented by others [23,24].

A recent study reported cost-effectiveness of shortening time to reports for bacterial culture from wounds, urine and blood and showed significant shortening of hospital stay and less antibiotic correlated with shorter time to reports, though no difference could be seen for blood cultures [25]. Another study showed that there was no effect on mortality when reports were given earlier, but resulted in shorter hospitalization for patients with GPC [23]. The mean time to report from time to positive signal was however 9.2 hours, which is considerably longer than the strategy proposed here and by others [24]. Another issue, although not in the scope of this study, is the time from when blood cultures are drawn to the time when the incubation starts in blood culture systems. With the centralization of clinical microbiology facilities, transports can be long and have a larger impact on time to first report than speeding up the work-flow in the laboratory. The time, from when the blood cultures are drawn until incubation starts, is measured and used as a medical quality index for logistics in microbiology diagnostics in Sweden. There are also discussions about when the laboratory should be staffed. In this study we only included positive blood cultures that signalled positive before ten in the morning in order not to interfere too much with the routine work. Some labs have already started to change working hours in order to get preliminary reports out before morning rounds. The effect of having more access (i.e. longer work-days and/or night shifts) to blood culture diagnostics may be beneficial for patient care, but has yet to be evaluated.

## Conclusions

A simple strategy to detect GPC directly from blood culture bottles gives reliable and cost effective reporting of > 90% of the most frequent findings within 6 hours after a positive blood culture. This enables faster administration of proper antimicrobial treatment and has potential to more rapidly ensure effective therapy for patients with bacteraemia. Moreover, close evaluation show that a direct TCT is a reliable and rapid test to identify *S. aureus*, but that the plasma species and volume of added material from blood culture bottles are important factors. For pneumococci and streptococci the rapid agglutination tests directly from positive blood cultures have very high positive predictive value but negative tests need to be repeated with colonies grown during 4–6 hours.

## Abbreviations

GPC: Gram-positive cocci; CoNS: Coagulase-negative staphylococci; MALDI-TOF: Matrix assisted laser desorption ionization-time of flight; TCT: Tube coagulase test; GAS/GBS/GCS/GFS/GGS: Group A/B/C/F/G streptococci; PYR: L-pyrrolidonyl-ß-naphthylamide; VRE: Vancomycin-resistant enterococci; MRSA: Methicillin-resistant *S. aureus*.

## Competing interests

The authors declare that they have no competing interests.

## Authors’ contributions

ML, HW, EL, FR, AM, KF contributed with laboratory work and for HW, EL, FR their work was used for diploma projects in their respective university formation. ML contributed in making figures and text. AW, TS contributed in expertise for design and revisions. LS initiated the project, financed and contributed to its completion. All authors have read and approved of the final manuscript.

## Pre-publication history

The pre-publication history for this paper can be accessed here:

http://www.biomedcentral.com/1471-2334/14/146/prepub
